# Innovative Reversed-Phase Chromatography Platform Approach for the Fast and Accurate Characterization of Membrane Vesicles’ Protein Patterns

**DOI:** 10.1021/acsptsci.4c00112

**Published:** 2024-04-11

**Authors:** Luca Nompari, Salvatore Sanna Coccone, Gian Luca Sardone, Alessio Corrado, Stefania Berti, Massimiliano Biagini, Michele Rovini, Claudia Magagnoli, Simona Cianetti, Serena Orlandini, Sandra Furlanetto, Riccardo De Ricco

**Affiliations:** † GSK, Technical Research and Development (TRD), Via Fiorentina 1, 53100 Siena, Italy; ‡ Department of Chemistry “U. Schiff″, University of Florence, Via U. Schiff 6, Sesto Fiorentino 50019, Florence, Italy

**Keywords:** vaccine characterization, reversed-phase chromatography, outer membrane vesicles, generalized modules for membrane antigens, experimental design, Neisseria meningitidis

## Abstract

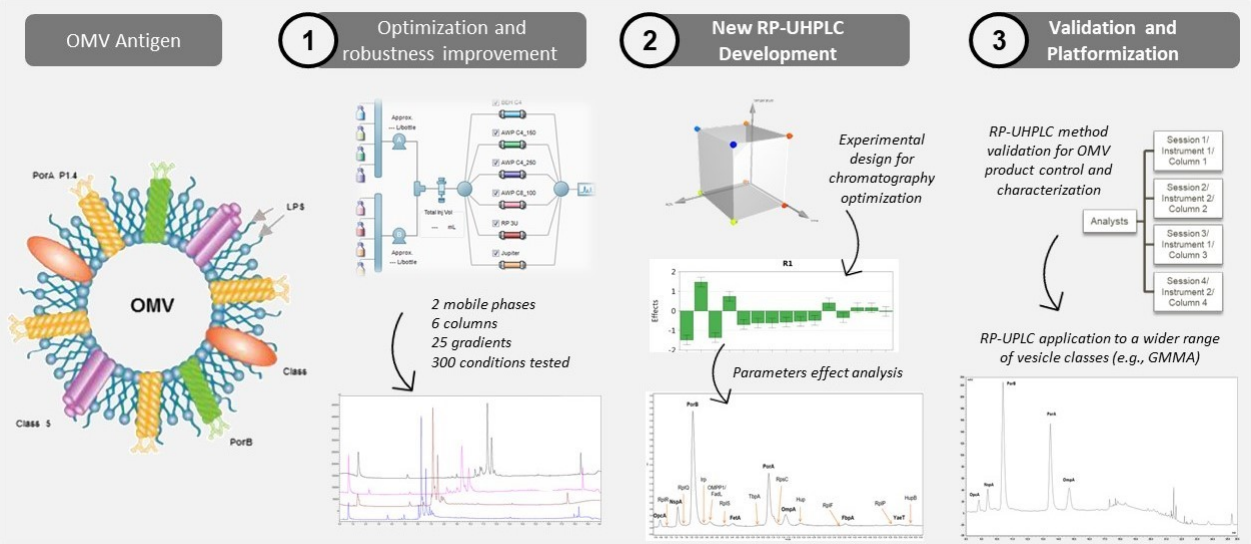

Outer membrane vesicles (OMVs) have been widely explored to develop vaccine candidates for bacterial pathogens due to their ability to combine adjuvant properties with immunogenic activity. OMV expresses a variety of proteins and carbohydrate antigens on their surfaces. For this reason, there is an analytical need to thoroughly characterize the species expressed at their surface: we here present a simple and accurate reversed-phase ultrahigh-performance liquid chromatography (RP-UPLC) method developed according to quality by design principles. This work provides an analytical alternative to the classical sodium dodecyl sulfate–polyacrylamide gel electrophoresis (SDS-PAGE) characterization. The higher selectivity and sensitivity of the RP-UHPLC assay allow for the identification of additional protein species with respect to SDS-PAGE and facilitate its precise relative abundance quantification. According to validation results, the assay showed high accuracy, linearity, precision, repeatability, and a limit of quantification of 1% for less abundant proteins. This performance paves the way for improved production campaign consistency while also being analytically simple (no sample pretreatment required), making it suitable for routine quality control testing. In addition, the applicability of the assay to a wider range of vesicle classes (GMMA) was demonstrated.

Over recent decades, outer membrane vesicles (OMVs) have been widely explored to develop candidate vaccines for bacterial pathogens.^1−3^ OMVs are able to combine adjuvant properties with immunogenic activity due to the concomitant presence of a variety of protein and carbohydrate antigens present on their surfaces. In addition, although OMVs have been directly derived from the pathogens themselves, they can also be engineered to express specific protein or carbohydrate antigens on a host source strain^4^ with limitless potential applications.

OMVs were first discovered in 1967 by studying the cell-wall structure of *Vibrio cholerae*,^5^ and the first clinical application was the immunization of patients with OMV generated from Meningococcal New Zealand B strain to control an outbreak of *Neisseria meningitidis* B (MenB) in New Zealand. Following positive results in controlling subsequent epidemics based on the MenB New Zealand strain, OMVs were successfully combined with 3 recombinant proteins, resulting in the now-commercial vaccine Bexsero (Bexsero is a trademark of the GSK group of companies), and the safety and effectiveness of which has been robustly demonstrated over the last 20 years.^6−8^

New vaccine candidates are currently under clinical evaluation that can be considered as a natural evolution of the OMV, also called generalized modules for membrane antigens (GMMA). GMMA are outer membrane vesicles engineered to produce an overvesiculating phenotype.^9,10^ These vesicles are easily produced and purified in a manufacturing setting and can be engineered to reduce the reactogenicity and improve the expression of specific proteins/saccharides. Their natural conformation resembles the bacterial pathogen surface^11,12^ such as OMV. This resemblance maintains pathogen-associated molecular patterns, such as lipopolysaccharides or lipoproteins, together with target antigens and provides, like OMV, a self-adjuvating effect.^13−15^

Due to their resemblance to the bacterial target surface, OMV/GMMA vaccines are, on the other hand, chemically and biochemically complex. Therefore, the associated analytical strategy to characterize the antigens exposed on their surfaces is critical, especially to establish a lot-to-lot consistency in different production campaigns. One of the most important critical quality attributes (CQA) of OMV/GMMA-based vaccines is their characterization in terms of the proteins (antigens) expressed on their surface (also called protein pattern).

The OMV/GMMA protein pattern is generally assessed by sodium dodecyl sulfate–polyacrylamide gel electrophoresis (SDS-PAGE)^16−18^ or more recently by liquid chromatography coupled with mass detection (LC-MS)^19−25^ although the applicability in GMP setting of these LC-MS methods remains limited.

With the aim of developing a fast and accurate assay to determine the pattern of protein antigens expressed on the surface of the OMV/GMMA complex, we herein report an alternative assay based on reversed-phase ultrahigh-performance liquid chromatography (RP-UHPLC). This new application has the potential to be of great importance in the field of characterization of the OMV/GMMA vaccines as it provides the implementation of a new concept and simpler way of working. In SDS-PAGE, the various proteins present on the OMV surface are discriminated based on mass. Although the resolution between different bands according to gel and buffer matrix can be quite efficient, different proteins with a similar weight might comigrate in the same band, reducing the specificity of the method. The technique usually requires coupling with MS characterization, especially to set up product specifications and criteria.

In RP-UHPLC, separation relies on protein-column affinity, giving a limitless approach to identify, and possibly characterize and quantify, each protein of the OMV with the use of a single UHPLC assay.

In this work, we report the translation of an SDS-PAGE protein pattern assay into an RP-UHPLC protein pattern assay. The method was applied to both OMV and GMMA of the same pathogen (*N. meningitidis* group B), demonstrating the applicability of the assay to both vesicle classes. *N. meningitidis* group B OMVs/GMMA were used as a case study; furthermore, the RP-UHPLC protein pattern method was successfully validated for MenB OMV.

## Experimental Section

### Drug Product Samples

The MenB OMV and MenB GMMA drug substances were provided by GSK as bulk solutions and were stored at 4 ± 2 °C. The bulks of the OMV and GMMA were injected without dilution or pretreatment. For additional details on MenB OMV and MenB GMMA production, please refer to ref (1).

#### Chemicals

Trifluoroacetic acid ≥99% CF_3_COOH (TFA, LC-MS, and HPLC grade), formic acid HCOOH (FA, LC-MS, and HPLC grade), and perfluoro-pentanoic acid 97% CF_3_(CF_2_)_3_COOH (PFPA, HPLC grade) were purchased by Sigma-Aldrich (Saint Louis, MO, USA). Methanol CH_3_OH ≥ 99.9% (HPLC grade) was purchased from Merck KGaA (Darmstadt, Germany). Acetonitrile (ACN) 99.8% (LC-MS grade) was purchased by Panreac (Radnor, PA, USA). Trypsin gold (Mass spectrometry grade) was purchased by Promega Corp. (Madison, WI, USA). Ultrapure water was produced by the Millipore Milli-Q system (Billerica, MA, USA). All solutions used were filtered on a nylon membrane of 0.22 μm porosity, using Nalgene clepsydra filters (Nalgene, Rochester, NY, USA).

### RP-UHPLC Equipment and Settings

LC columns tested are Acquity RP-C4 BEH 300 Å, 1.7 μm, 2.1 × 150 mm (BEH C4) from Waters Corp. (Milford, MA, USA); Aeris WIDEPORE C4 200 Å, 3.6 μm, 4.6 × 150 mm (AWPC4_150); Aeris WIDEPORE C4 200 Å, 3.6 μm, 4.6 × 250 mm (AWPC4_250); Aeris WIDEPORE XB-C8 200 Å, 3.6 μm, 4.6 × 100 mm (AWP C8); Jupiter C5 300 Å, 5 μm, 2.0 × 150 mm (Jupiter) from Phenomenex (Torrance, CA, USA); ProSwift RP-3U monolithic column, 4.6 × 50 mm (RP-3U) from Thermo-Fischer Scientific (Waltham, MA, USA); and Acquity BEH C8 1.7 μm 2.1 × 150 mm (BEH C8) from Waters Corp. (Milford, MA, USA).

The chromatographic configurations used for columns scouting and experimental designs were the NexeraX2 method scouting UHPLC series 30 system equipped with LC-30AD pump, DGU-20A5R degasser unit and LPGE-unit, SIL-30AD autosampler, CTO-20AC oven with 180 μL mixer, FCV-34AH UHPLC switching valve, SPD-M30A PDA detector (detection wavelength 280 nm; 4 nm resolution), and highly sensitive flow-cell (85 mm; 9 μL) from Shimadzu Corp. (Kyoto, Japan).

The RP-UHPLC method development activities and settings are more thoroughly discussed in the result sections 2.1, 2.2, 2.3, and 2.4. The RP-UHPLC final settings for the OMV/GMMA protein pattern method are herein reported: Acquity H-Class Bio UHPLC system (equipped with bioQSM, bioSM-FTN, and ACQ-PDA), 214 nm wavelength detection through an Acquity PDA analytical flow cell (FC: 10 mm–500 nl). Separation occurs through an Acquity BEH C8 1.7 μm 2.1 × 150 mm column with temperature control at 70 °C (system preheater active). Injection volume of 20 μL of undiluted OMV/GMMA bulks (by using a 50 μL extension loop, MP35N), and autosampler temperature controlled at 12 °C. The components separation occurred in a 0.55 mL/min flow rate: 1 min isocratic equilibration (30% ACN: 0.1% TFA: 69.9% water) postinjection, first elution step of 15 min with a linear gradient up to 40% ACN: 0.1% TFA: 59.9% water mixture. The second elution step took 5 min linear gradient up to 90% ACN: 0.1% TFA: 9.9% water mixture and a final column stripping for 4 min in isocratic elution (90% ACN: 0.1% TFA: 9.9% water).

### Computations and Software

LabSolution Version 5 software equipped with a Method Scouting start-up kit and licensed by Shimadzu Corp. was used for the NexeraX2 UHPLC instrument control and for the chromatographic data computation of purity and resolution of each chromatographic peak. Chromatographic resolutions (*R*) between two adjacent peaks were calculated using the retention times (*t*_R_) and the peak widths at half height (*w*), according to the following formula:
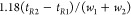
1

Purity of each chromatographic peak (*A*%) was calculated by the percentage ratio of the peak area (*A*_i_) with respect to total area (ATOT), according to the following formula:
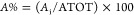
2

Peak capacity was controlled by counting the number of resolved peaks for each chromatographic run.

Empower 3 (Waters Corp.) software was used for RP-UHPLC fine-tuning, method lock, and validation.

BioPharma Finder Version 2.0 (Thermo Scientific) and PEAKS Studio Version 8.0 (BioInformatics Solutions) software were employed for computations of intact mass and peptide mapping by MS, respectively.

### SDS-PAGE

OMV proteins were separated on SDS-PAGE based on a mass loading of 5 μg per lane. Gels were stained with Coomassie Blue R-250 and scanned with Scanner Perfection V750 pro running and Labscan V3.00 (Epson Corp.) the software. Images were analyzed with an ImageMaster 1D elite V3.01 (GE Healthcare Life Sciences).

Deeper details about SDS-PAGE and the procedure for in-gel trypsin digestions of the protein bands are published by Tani et al.^19^ Protein identification was performed by LC-MS/MS as described in the Supporting Information.

## Results

The OMV component of the Bexsero vaccine (4CMenB, produced by GSK) is a stable colloidal suspension that consists of small, membranous, spherical vesicles, in which the native complex antigen composition of the subcapsular cell surface of *N. meningitidis* serogroup B is highly preserved.^26^ The OMV component contains several proteins of the outer membrane: PorA and PorB are the main proteins expressed, but other minor outer membrane proteins such as OmpC, FetA, OmpA, FbpA (and many others), and lipo-oligosaccharides (LPS) are also present.^26−28^

**Figure 1:**
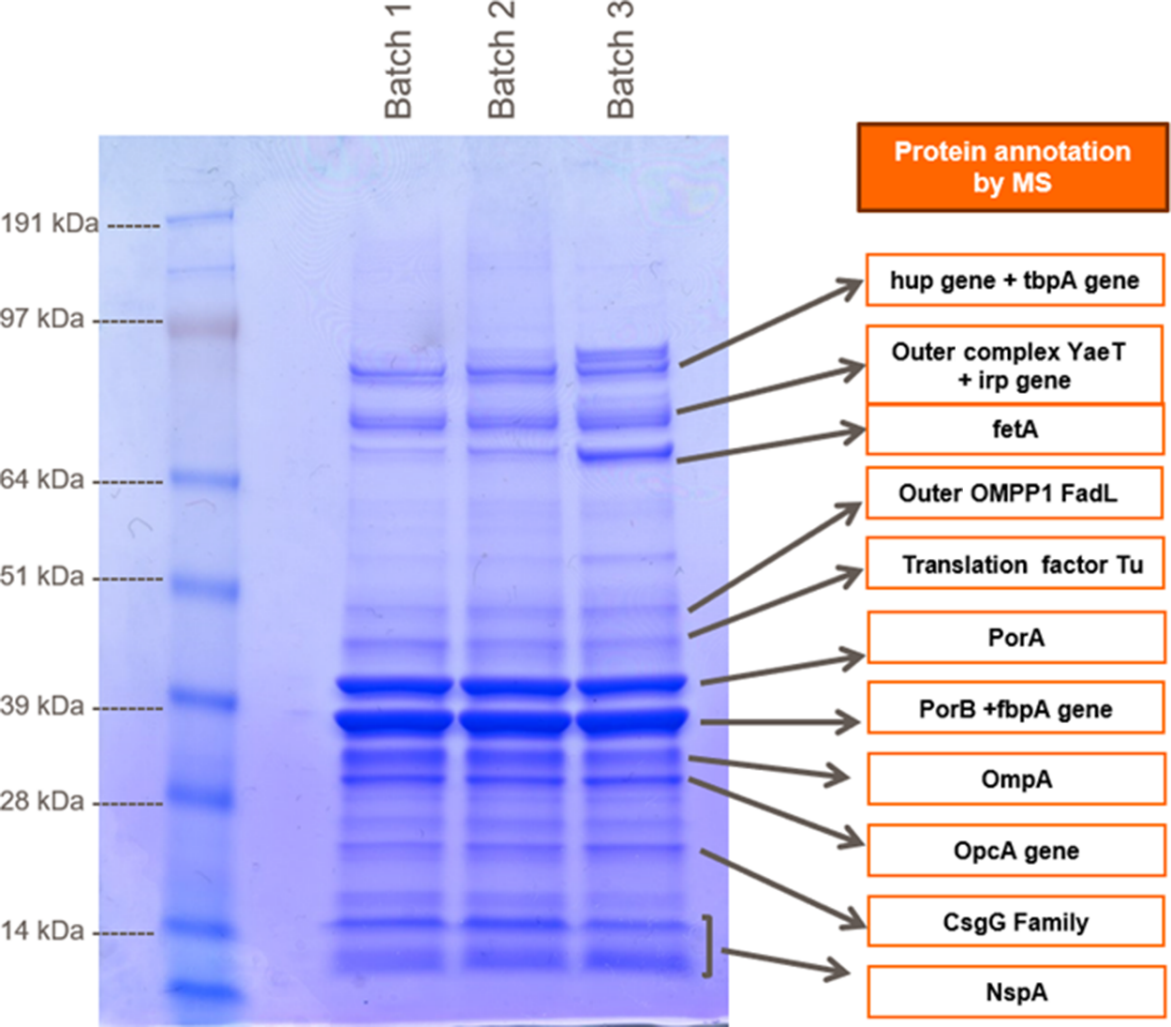
OMV protein pattern by SDS-PAGE assay for testing and control of the main components: PorA, PorB + FbpA comigration, OmpA, NspA, OpcA, FetA, and YaeT. Protein identification was obtained by in-gel digestion followed by LC-MS/MS of the major Coomassie Blue-stained bands. Batches 1, 2, and 3 are three different preparations of the OMV (batch consistency check).

The analytical method classically applied for the quality control of OMV protein’s identity and purity of the OMV protein is the densitometry SDS-PAGE assay. Figure 1 shows the typical OMV SDS-PAGE profile, which is based on the separation between surface-expressed species based on different sizes/charges.

SDS-PAGE is a very robust and simple test that is well established in research and quality control for the characterization of complex products. As proteins are grouped according to their molecular weight, however, it is not possible to quantify and discriminate the specific contribution of proteins with similar molecular weights within the same band. This may limit complete characterization and reduce product knowledge, with less information on each individual protein within the OMV/GMMA vesicles. In addition, SDS-PAGE requires several sample preparation steps (denaturation, use of standardized staining solutions, and knowledge of how to handle the gel for scanning, as well as how to identify the bands).

To develop a more advanced and precise method to replace the use of SDS-PAGE, liquid chromatography (RP-UHPLC) was explored. In this context, the screening of commercially available UHPLC reverse-phase columns as well as method refinements was conducted according to analytical Quality by Design (AQbD) principles. This resulted in the development of a fast and efficient method.^29−34^

RP-UHPLC was selected not only to increase the precision and throughput of the method but also to obtain a more accurate characterization and quantification (as relative abundance) of each detectable protein present in the OMV respect to the SDS-PAGE.

The main goals of the new method can be summarized as (i) revealing at least the same number of outer membrane proteins detected by the SDS-PAGE assay; (ii) being selective and specific to PorB and FbpA proteins (not resolved in SDS-PAGE assay); (iii) improving method throughput and robustness; and (iv) complying with general validation requirements according to ICH-Q2(R1) guideline.^4^

### Column Screening

For the initial screening of the possible RP-UHPLC columns to be used for the OMV protein pattern, the drivers considered were the capability to separate the higher number of peaks, revealing at least eight of the same number of proteins identified by SDS-PAGE (peak capacity) and a baseline resolution between peaks (selectivity).

The screening exercise took into account the following parameters of different stationary phases (Table 1): (i) chemistry (RP-C4, RP-C5, and RP-C8), (ii) support (silica and PSDVB particles), (iii) column length (from 50 up to 250 mm), (iv) particle porosity (from 200 Å pores up to 5.2 μm monolith), and (v) column technology (pore particles, solid core, and monolithic).

**Table 1:** Reverse-Phase Columns Were Selected for Scouting

column	supplier	abbreviation	stationary phase chemistry	support (particles)	length (mm)	particle porosity (Å)	technology
Acquity RP-C4 BEH 300 Å, 1.7 μm, 2.1 × 150 mm	Waters Corp.	BEH C4	C4	silica	150	300	pore particles
Aeris WIDEPORE C4 200 Å, 3.6 μm, 4.6 × 150 mm	Phenomenex	AWPC4_150	C4	silica	150	200	solid core
Aeris WIDEPORE C4 200 Å, 3.6 μm, 4.6 × 250 mm	Phenomenex	AWPC4_250	C4	silica	250	200	solid core
Aeris WIDEPORE XB-C8 200 Å, 3.6 μm, 4.6 × 100 mm	Phenomenex	AWP C8	C8	silica	100	200	solid core

The NexeraX2 system allowed for the simultaneous screening of each of the six columns evaluated in a single analytical session, including the simultaneous testing of different mobile phases and linear gradients from low to high organic concentrations. The organic phases tested in the one-factor-at-time experiment (OFAT) were ACN (acetonitrile) + 0.1% (v/v) TFA (trifluoroacetic acid) and MeOH (methanol) + 0.1% (v/v) TFA.

**Figure 2:**
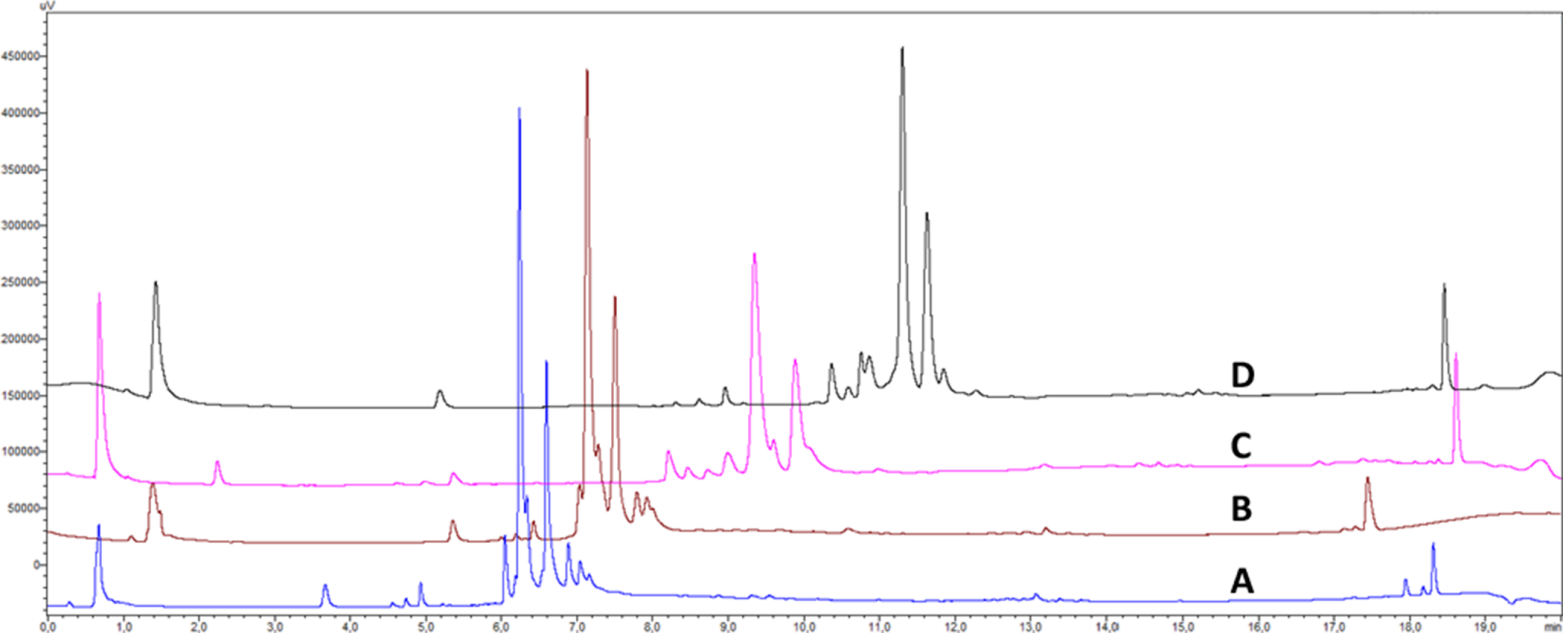
Best chromatographic profiles of RP-UHPLC scouting. Injection of 5 μL MenB OMV bulk for each run, without sample dilution and treatment. (A) Blue line is the BEH C4 column eluted in an acetonitrile/water/TFA mixture, (B) brown line is the AWP C8 column eluted in an acetonitrile/water/TFA mixture, (C) pink line is the AWP C8 column eluted in a methanol/water/TFA mixture, and (D) black line is the BEH C4 column eluted in a methanol/water/TFA mixture.

The chromatographic initial and final organic concentrations studied for the linear gradient were from 5 to 45% (ACN + 0.1% (v/v) TFA) as the starting ramp concentration and from 100 down to 60% for the final ramp concentration. A 2 min washing step and 2 min of re-equilibration (under starting conditions) were included at the end of the chromatography. A summary of the most representative results obtained is shown in Figure 2: BEH C4 (A and D) and AWP C8 (B and C) columns showed the highest peak capacity and selectivity, in alignment with initial expectations for all the different chromatographic conditions tested. For this reason, these 2 columns were further studied in an additional DoE (design of Experiment) screening.

### Risk Assessment of Potential Critical Method Parameters (pCMPs)

**Figure 3:**
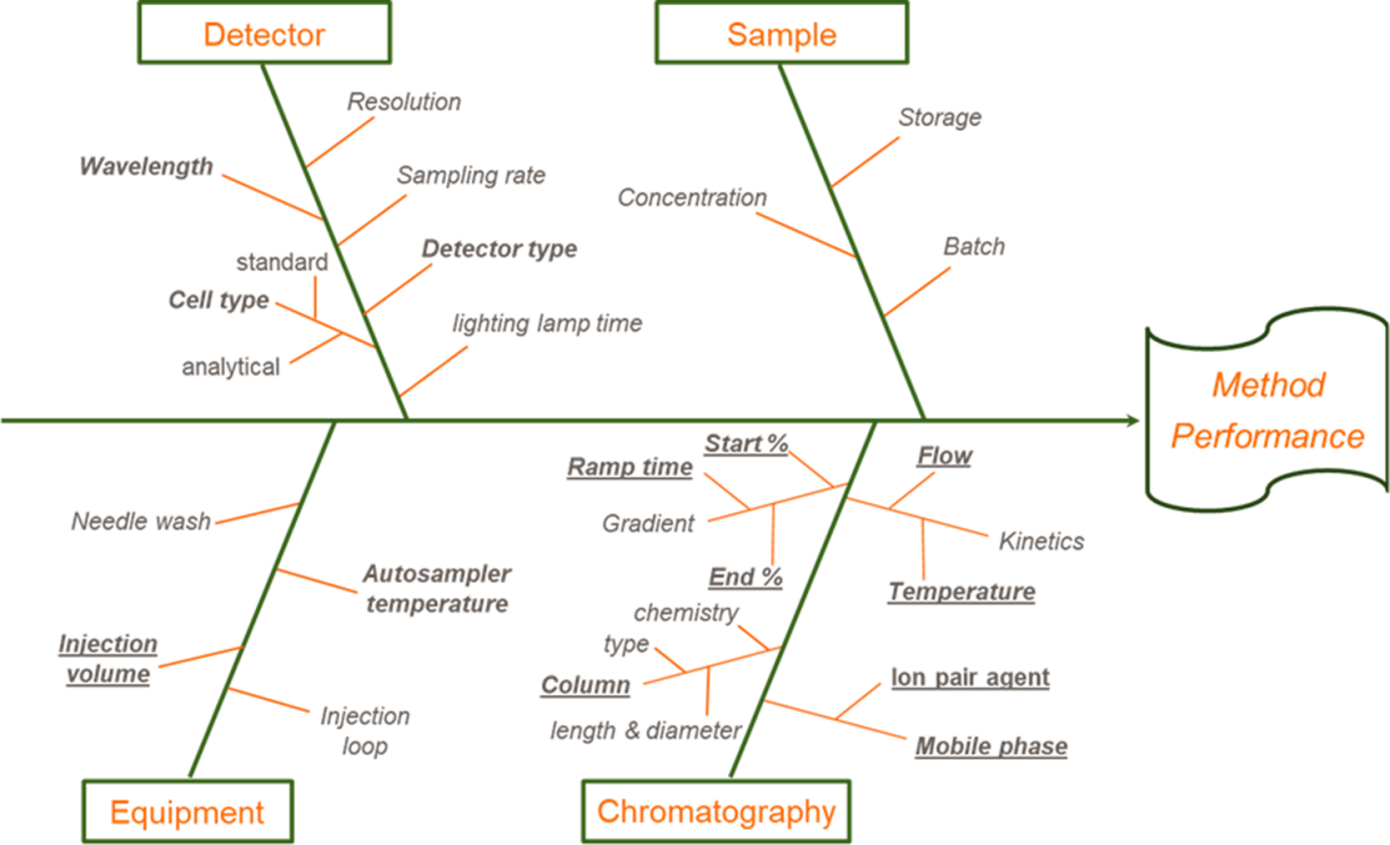
Ishikawa diagram for the RP-UHPLC OMV protein pattern. pCMPs already locked (to not be further explored) are identified in bold, pCMPs to be explored are identified in bold and underlined, and all the other parameters are not considered as pCMPs.

Following a preliminary method/column screening, an assessment based on process mapping^9^ and an Ishikawa diagram^10,29^ was performed to further evaluate the variability of each method and its impact on the reportable values (peak capacity and selectivity). pCMPs identified were mitigated through a risk assessment.^30^ The risk assessment exercise is reported in the Supporting Information, while the Ishikawa diagram is reported in Figure 3. The pCMPs, highlighted in bold in Figure 3, were preliminarily investigated in the initial column screening and not explored thereafter. All of the other pCMPs, highlighted in bold and underlined in Figure 3, were studied in an additional DoE to find the optimal conditions.

### Screening Experimental Designs

The gradient shape reported in Figure S1 was used as the starting point to optimize the chromatographic selectivity, according to the attributes highlighted in Figure 3 (bold + underlined). The pCMPs to be investigated by experimental design were mobile phase type (MP: *acetonitrile, ACN*; *methanol, MeOH*), column type (COL: *BEH C4* and *AWP C8*), column temperature (TEMP: *50; 60; 70;* and *80 °C*), injection volume (VOL: *2; 6;* and *10 μL*), time for gradient ramp A (RAMP A: *2; 4; 6;* and *10 min*), time for gradient ramp B (RAMP B: *2; 6; 10;* and *15 min.*), time for gradient ramp C (RAMP C: *2; 4; 6;* and *10 min.*), starting organic % of ramp B (%A: *20; 30; 40;* and *50%*), ending organic % of ramp B (%B: *40; 50; 60;* and *70%*), mobile phase flow (FLOW: *0.3; 0.5; 0.7;* and *0.9 mL/min.*), and ion-pairing agent (ION: *formic acid, AF; trifluoroacetic acid, TFA; and pentafluoro propionic acid, PFPA*). Considering the high number of method parameters, two sequential DoEs were performed (DOE_1 and DOE_2) using MenB OMV bulk material.

The main drivers of the DoE evaluations were the total numbers of peaks (*N*), the number of peaks in the gradient ramp B (nB), the PorB capacity factor (*K′*), and finally, the resolution between PorB and PorA outer membrane proteins (*R*1). All of those parameters cover the initial objectives of obtaining an optimal peak capacity and selectivity.

**Figure 4:**
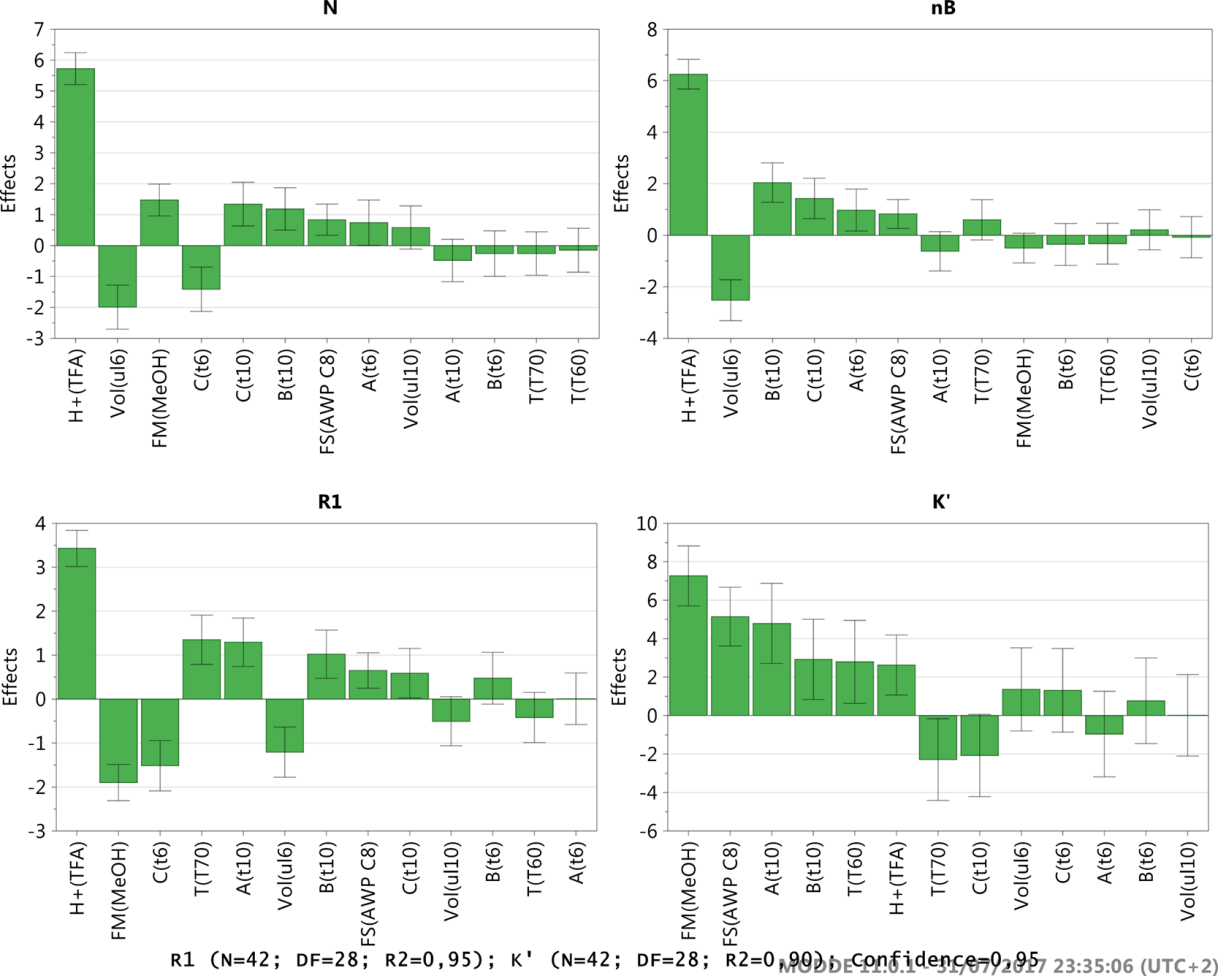
D-Optimal DoE_1-graphic effects analysis. The assessment of parameter effect for *N*, nB, *R*1, and *K′* outputs is represented by a box and whiskers plot. The green bars represent the effects of each tested parameter from level −1 to level +1. The gray bars report the error. If the error contains zero, the parameter does not have a significant effect. On the contrary, the parameter/level has a positive or negative effect on the response under consideration.

In the first 20-run asymmetric (2^3^3^5^//20) D-optimal design (DoE_1), the effects of MP, TEMP, VOL, COL, ION, RAMP A, RAMP B and RAMP C were investigated (Table S1). A center point was included in the design and each run was duplicated to obtain a reliable estimation of the experimental variance (42 total runs, G-efficiency: 81%). The best conditions identified were: COL: AWP C8; TEMP: 70 °C; SOL: ACN; RAMP A: 6 min.; RAMP C: 10 min.; and INJ: 10 μL. The DoE_1-graphic effects analysis is reported in Figure 4.

**Figure 5:**
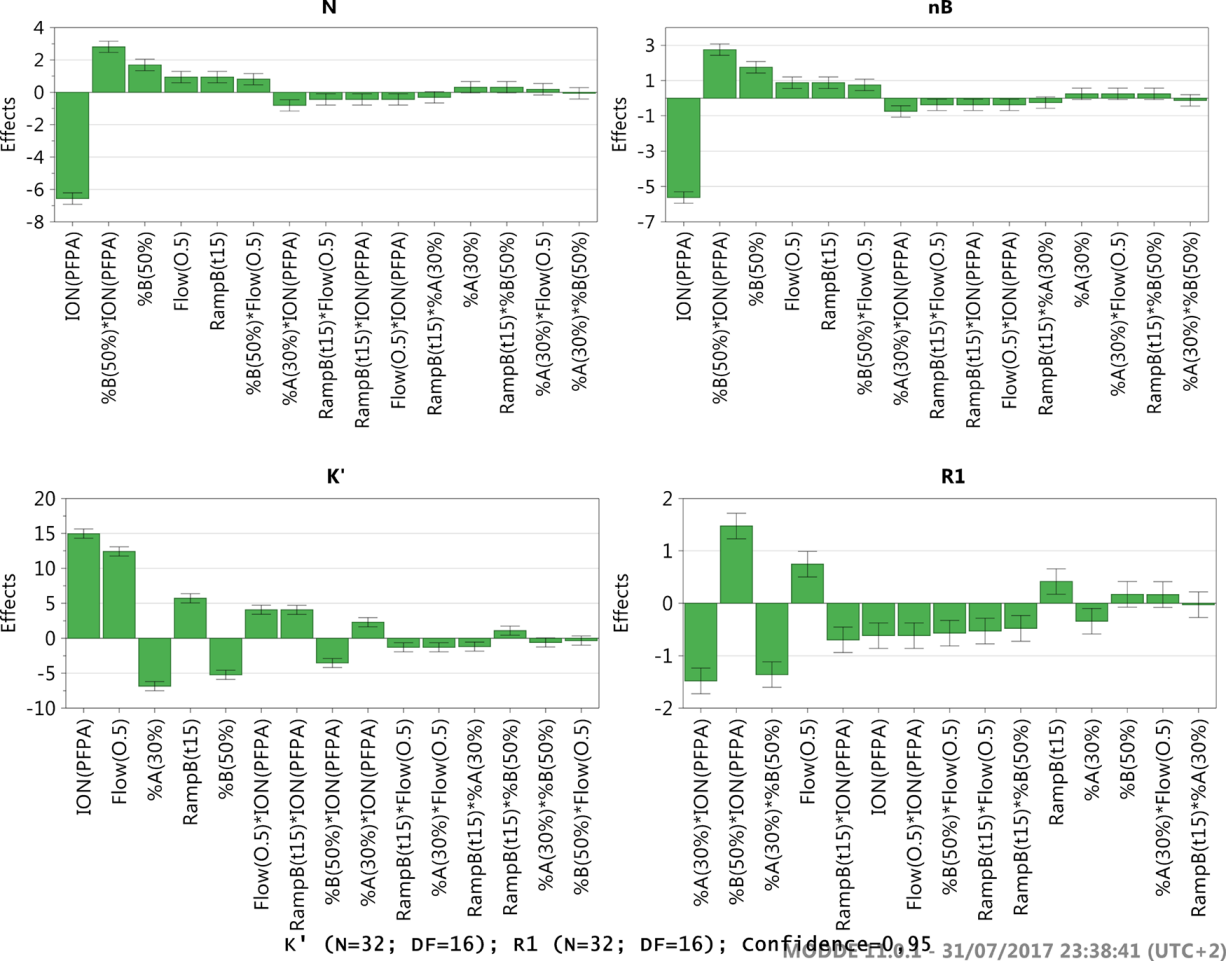
Fractional factorial DoE-graphic effects analysis. The assessment of parameters effect for *N*, nB, *R*1, and *K′* outputs is represented by a box and whiskers plot. The green bars represent the effects of each tested parameter from level −1 to level +1. The gray bars report the error. If the error contains zero, the parameter/level does not have a significant effect. On the contrary, the parameter has a positive or negative effect on the response under consideration.

Following the results of DoE_1, a new 16-run fractional factorial (2^5^//16) resolution V design was performed to better investigate the effects of RAMP B and ION, with the addition of %A, %B and FLOW factors, for the selected critical method attributes (CMAs) (Figure 5). Each run was duplicated to obtain a reliable estimation of the experimental variance (Table S2). From the DoE_2-graphic effects analysis (Figure 5) the optimal conditions identified were: ION, TFA; RAMP B, 15 min; %A, 30%; %B, 40%; and FLOW, 0.5 mL/min.

**Figure 6:**
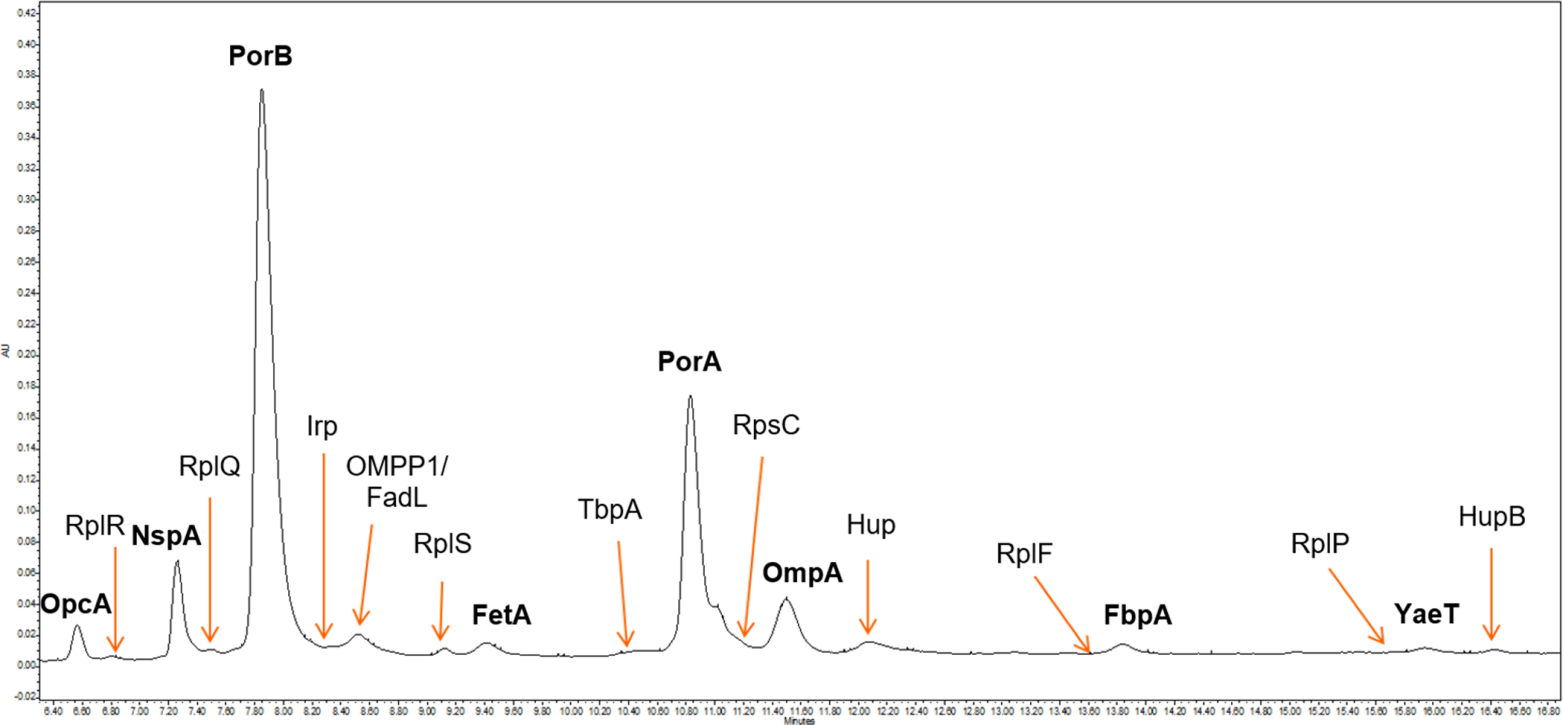
RP-UHPLC protein pattern profiling of MenB OMV by Acquity BEH C8, 1.7 μm, 2.1 × 150 mm.

Following the outcome of the DoEs, further method refinements were performed with the OFAT approach leading to the final RP-UHPLC chromatography profiling for the OMV protein pattern as shown in Figure 6.

**Figure 7:**
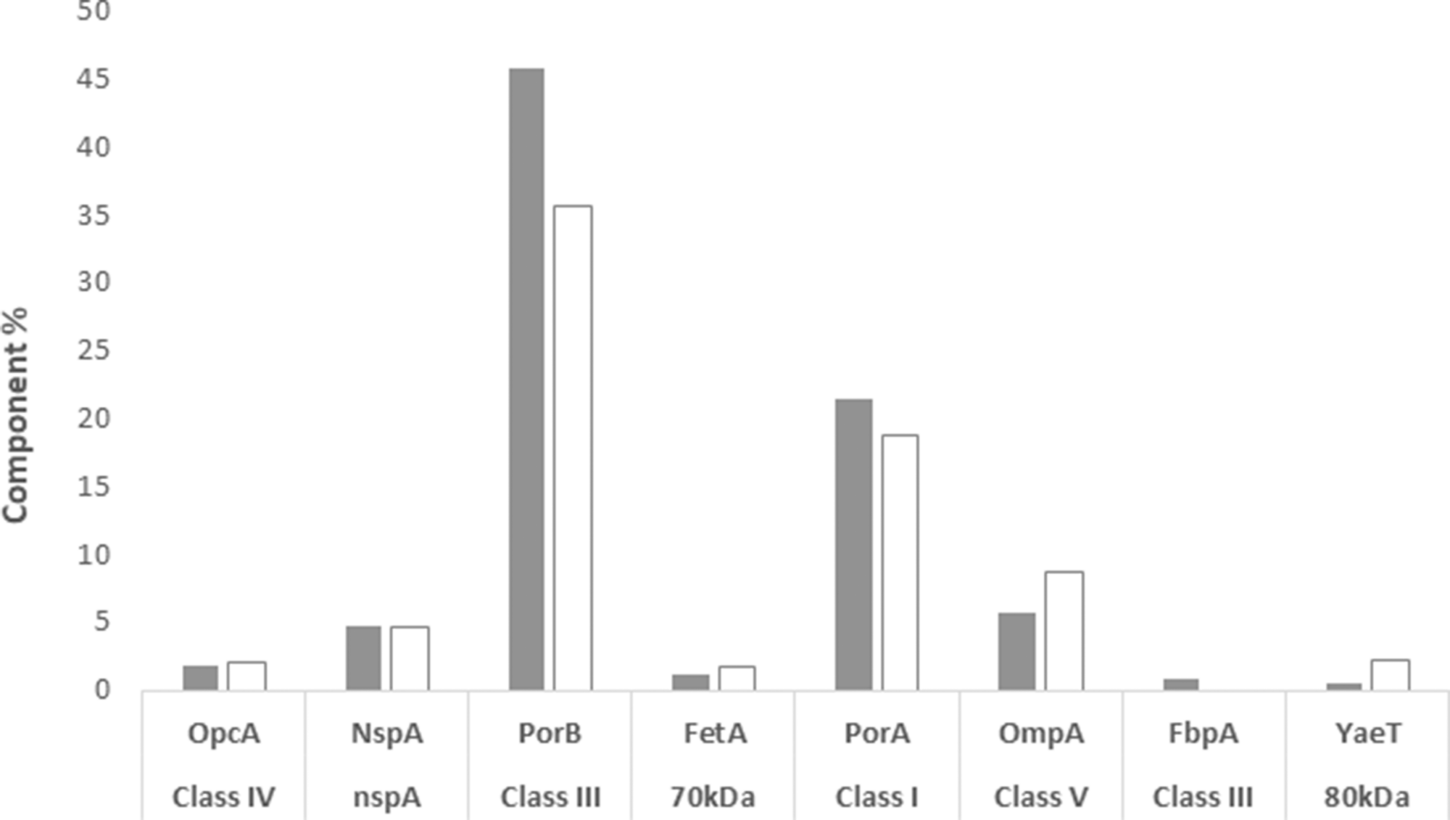
MenB OMV protein pattern comparison between RP-UHPLC (gray bars) and SDS-PAGE (blank filled bars) assays. Additional details on RP-UHPLC method performances are reported in Table 2. The SDS-PAGE assay variability is reported in the literature (18).

The identified conditions satisfied the objectives defined for the development of the following method:

1. The same proteins quantified using SDS-PAGE can be quantified using the new RP-UHPLC method.
2. PorB and FbpA proteins are selectively separated and identified.
3. Additional proteins (not revealed in SDS-PAGE) are detected and identified.
4. The throughput of the assay is increased, no pretreatment is required.

LC-MS analysis was used to confirm the identity of the proteins revealed in the OMV by the new assay, confirming the selectivity of the new RP-UHPLC method for the protein pattern investigated. The characterization and identification of the peaks within the chromatogram, reported in Figure 6, were performed by applying two different MS approaches: average molecular weight determination (by LC-MS intact mass determination) and identity confirmation (by LC-MS/MS-based peptide mapping). Details of the MS characterization techniques are reported in the Supporting Information and in ref (35).

Both techniques confirmed the identity of all of the peaks identified and showed differences between protein variants present in each of the peaks. All the information regarding the approaches used and of the results obtained are reported in the Supporting Information.

### Method Fine-Tuning and Lock

Following the routine implementation of the Aeris WIDEPORE XB-C8 200 Å, 3.6 μm, 4.6 × 100 mm column (by Phenomenex), reproducibility issues were observed due to batch-to-batch resin variability. To increase method robustness, the optimized chromatography conditions were tested on a different column routinely used in quality control (QC) analysis: the Acquity UHPLC BEH C8, 1.7 μm, 2.1 × 150 mm (by Waters Corp.). According to the new column properties, the injection volume was adjusted to 20 μL. A representative chromatogram obtained with the final method is reported in Figure 6: peaks marked in bold are the eight main OMV proteins (PorA, PorB, OmpA, FetA, NspA, OpcA, FbpA, and YaeT) monitored by SDS-PAGE; nonbolded peaks are the additional proteins detected only by the RP-UHPLC assay. Three different column/resin batches were tested and compared (Table S3). The good results obtained confirmed the high reproducibility of the BEH C8 stationary phase and comparable performance in terms of peak capacity and selectivity with respect to the previously selected column (Aeris WIDEPORE XB-C8 200 Å, 3.6 μm, 4.6 × 100 mm). The identity of each RP-UHPLC peak was assessed using mass spectrometry (see Supporting Information for additional details).

Finally, SDS-PAGE and RP-UHPLC performances were compared (Figure 7). Results comparing the species identified by SDS-PAGE versus results obtained by RP-UHPLC are aligned, although, as expected, the chromatographic assay showed better performance in terms of accuracy, linearity, and precision (data not shown).^31^ For these reasons, the new RP-UHPLC protein pattern performances were further evaluated through method validation in a GMP setting.

**Figure 8:**
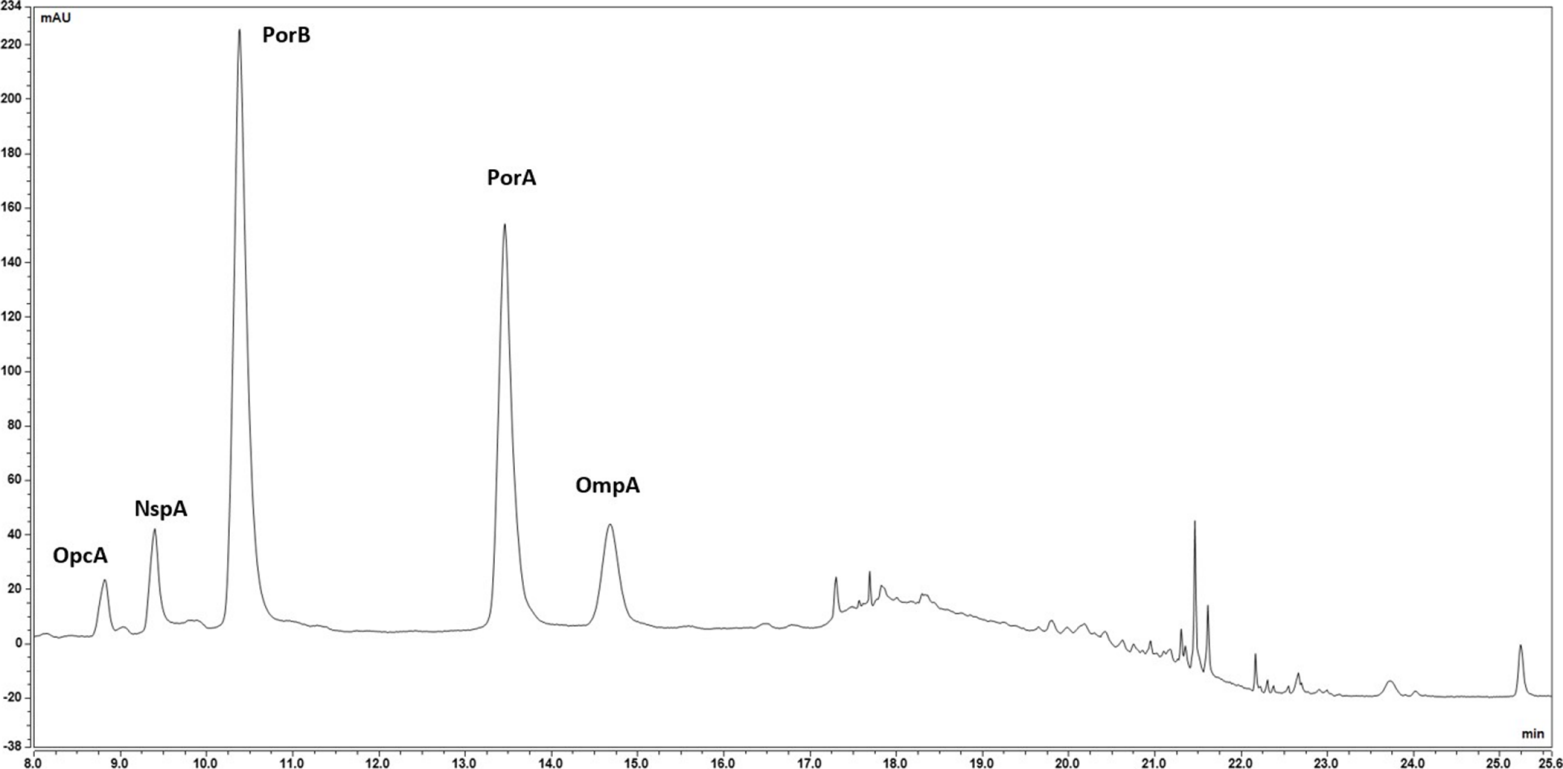
RP-UHPLC protein pattern profiling of MenB GMMA. The identity of each RP-UHPLC peak was assessed using mass spectrometry (data not shown).

### Method Validation

Following a comparison of the performances of the two assays, the new RP-UHPLC test was validated according to ICH-Q2(R2) requirements. A summary of the results obtained during method validation is reported herein, demonstrating the high precision and reproducibility of the high-throughput RP-UHPLC assay. The method demonstrated better performance for the most abundant proteins (PorA and PorB, present at ∼20 and ∼30%, respectively) and overall, on the sum of the most abundant proteins (herein referred to as MPRA: major proteins relative amount obtained as the sum of PorA + PorB + OpcA + NspA + FetA + OmpA) (Table 2). A lower assay performance was observed for less abundant proteins (OpcA, NspA, FetA, and OmpA).

**Table 2:** Validation Performances of the OMV Protein Pattern RP-UHPLC Method

parameter	PorA	PorB	OpcA	NspA	FetA	OmpA	MPRA^a^
accuracy (%)	[98, 105]	[99, 105]	[98, 104]	[−90, 105]	[83, 109]	[95, 102]	[99, 104]
intermediate precision	2–3%	2–4%	2–4%	3–13%	11–20%	3–9%	2–3%
repeatability	0.6%	0.2%	2%	1%	0%	2%	0.2%
sample linearity	*R*2 > 0.998	*R*2 > 0.998	*R*2 > 0.998	*R*2 > 0.998	*R*2 > 0.998	*R*2 > 0.998	*R*2 > 0.998
specificity	specific for matrix components						
LOQ	minimum relative concentration of a protein measured: 1%						
range	test applicable on an OMV protein concentration range of 350–1460 μg/mL						

a MPRA: major proteins relative amount obtained as sum of PorA + PorB + OpcA + NspA + FetA + OmpA.

### General Applicability (Platformization) of the RP-UHPLC Protein Pattern Assay to GMMA Vesicles

The applicability of the assay to different classes of vesicles was tested on GMMA candidates, which can be considered as the natural evolution of classical OMV (Figure 8).

As shown in Figure 8, although a minor shift in the retention time of the most abundant proteins was observed (which may be related to different matrices of the analyte), a similar profile was obtained for the MenB GMMA construct with respect to that of the natural OMV. MS characterization confirmed the identity of the peaks (according to the peak label), which is consistent with that of the OMV antigen. Those data confirmed the platform applicability of the assay to both the OMV and GMMA constructs.

## Discussion and Conclusions

The translation of a classical SDS-PAGE protein pattern assay to assess the relative percentage of the most abundant protein expressed on the surface of the OMV vesicles was demonstrated through an RP-UHPLC assay. The RP-UHPLC assay was shown to be a precise and simple assay for the determination of the proteins expressed on membrane vesicles, with an improved throughput as compared to the current technique. The new RP-UHPLC method satisfied the objectives defined at the beginning of method development: (i) at least the same proteins detected by SDS-PAGE are identified by the new method; (ii) additional proteins not revealed in SDS-PAGE are detected and identified; (iii) the PorB and FbpA proteins selectivity is improved (not resolved with SDS-PAGE assay), and each antigen is independently identified; and (iv) the throughput of the assay is increased, no pretreatment is required.

So far, only mass-spectrometry-based methods^19−22^ or 2D-PAGE have been reported in the literature as alternatives to the classical SDS-PAGE to obtain rapid and robust characterization of MenB OMV structure composition, especially for formulated drug products containing aluminum adjuvants.^21,26^ The implementation of such complex techniques, however, has limited their application in a quality control (QC) environment (especially in terms of data evaluation and interpretation), where fast and simple assays are required. Nevertheless, considering the variety of proteins present on the OMV/GMMA surfaces and their critical role in immunogenicity, it is fundamental to address and control their relative abundance in a consistent and precise manner through a user-friendly assay. Our new approach, while also being simple and easily implemented in QC as a routine test, allows for the identification of a larger number of proteins with respect to SDS-PAGE: in addition to the eight most abundant proteins revealed by SDS-PAGE, 11 additional proteins were identified by the RP-UHPLC assay reported here (Figure 7). The different mechanism of separation of proteins present in the OMV/GMMA allows for improved batch-to-batch consistency. In addition, the lead time and the complexity in both sample preparation and data elaboration are significantly reduced with respect to classical SDS-PAGE.

Solid knowledge of MODR (the operating range for the critical method input variables that produce results that consistently meet the goals set out in the ATP) permits flexibility in various inputs of method parameters to provide the expected method performance criteria and method response: once a (platform) method has been qualified for a product modality, implementation of the subsequent molecules for the same product modality can be supported by an abbreviated method verification exercise. For example, in the case of a new formulation of the product, evaluating the effects of variation of a potential critical method parameter within the boundaries of the established MODR would allow for optimization of the new required conditions to satisfy the ATP, maintaining the analytical knowledge previously collected.^36−38^

According to these principles, the method was then shown to be suitable for different vesicle candidates (OMV and GMMA), with similar profiles and the same levels of peak resolution obtained. In particular, by comparing through this innovative RP-UHPLC assay OMV and GMMA of the same bacterial target (MenB), it was possible to ascertain that the most abundant proteins are conserved between the two different production systems. This relative assay therefore makes it possible to compare the relative abundance of different types of vesicles.

Considering the increased interest over recent decades for the development of new vaccines based on OMV/GMMA candidates for many different vaccine targets, this new analytical method has the potential to be of great relevance for current and future vaccine programs. The relevance of this development is related not only to the increased throughput of the assay and the reduced lead time but also to the improved characterization of the proteins expressed at the surface of the vesicle. Furthermore, it enables a deeper knowledge of the product and of its reproducibility, granting a higher control over the production campaigns of the OMV/GMMA and product life-cycle management.

## Supporting Information Available

Additional information on the shape of the elution gradient used to optimize the chromatography and DoE parameters annotation; Gaussian charge-states distribution of PorA protein; opcA and nspA proteins; porB and fetA proteins; ompA and porA proteins; and of fbpA and yaeT proteins; raw data and experimental design of DoE-1 and of DoE-2 studies; batch-to-batch chromatographic column data reproducibility; molecular weight determination and peptide mapping analysis for OMV proteins; additional information on MS assay configuration: LC-MS analysis and peptide mapping method details; and details of the MS characterization techniques and corresponding results (PDF)
