# Supplementary material for: Innovative Reversed-Phase Chromatography Platform Approach for the Fast and Accurate Characterization of Membrane Vesicles’ Protein Patterns
Source: ACS Pharmacol Transl Sci. 2024 Apr 11;7(5):1584–94. doi: 10.1021/acsptsci.4c00112 (PMC11091982; doi:10.1021/acsptsci.4c00112)
Supplement: Supplementary file 1 — pt4c00112_si_001.pdf [file pt4c00112_si_001.pdf]

Supporting Information

An innovative Reversed Phase Chromatography platform approach for the fast and accurate characterization of membrane vesicles' protein patterns

Luca Nompari[1], Salvatore Sanna Coccone[1], Gian Luca Sardone[1], Alessio Corrado[1], Stefania Berti[1], Massimiliano Biagini[1], Michele Rovini[1], Claudia Magagnoli[1], Simona Cianetti[1], Serena Orlandini[2], Sandra Furlanetto[2] and Riccardo De Ricco[1]*

1 GSK, Technical Research and Development (TRD), Via Fiorentina 1, 53100 Siena, Italy.

2 Department of Chemistry "U. Schiff", University of Florence, Via U. Schiff 6, 50019 Sesto Fiorentino, Florence, Italy.

* Riccardo De Ricco riccardo.x.de-ricco@gsk.com

Figure S 1 **RP-UHPLC starting gradient used to optimize the chromatography.**

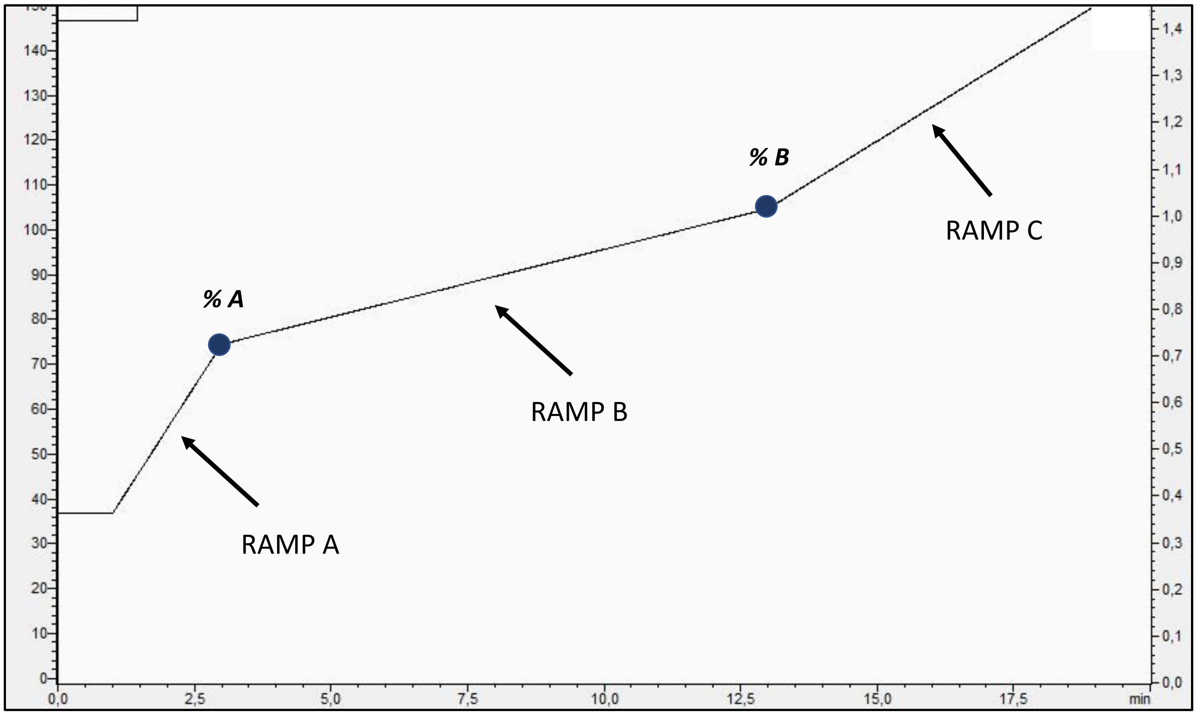

Ramp A is the ramp time taken to increase the initial column equilibration and binding condition to percentage concentration A (%A). %A is the starting organic condition to define the gradient of ramp B. %B is the final organic condition to define the gradient of ramp B. Ramp B is the ramp time taken to go from %A to %B. Ramp C is the ramp time taken to get the final elution mixture.

Table S 1 D-Optimal screening experimental design (DoE-1)

**Exp No**	**MP**	**ION**	**TEMP** °C	**VOL** µl	**COL**	**RAMP A** min.	**RAMP B** min.	**RAMP C** min.	**R_1**	**N**	**nB**	**K'**
1	ACN	TFA	60	6	BEH C4	2	2	2	2,353	11	4	2,600
2	ACN	AF	50	2	AWP C8	6	2	2	1,868	8	3	3,432
3	MeOH	TFA	50	10	AWP C8	2	6	2	2,231	15	7	11,629
4	ACN	TFA	70	2	BEH C4	10	6	2	8,351	13	9	3,587
5	MeOH	AF	70	6	BEH C4	6	10	2	0,044	8	1	5,513
6	MeOH	AF	60	10	AWP C8	10	10	2	0,691	10	2	16,956
7	MeOH	TFA	70	10	BEH C4	6	2	6	1,596	14	8	5,684
8	ACN	AF	60	10	AWP C8	10	2	6	0,678	7	2	3,568
9	ACN	AF	70	6	AWP C8	2	6	6	0,001	5	1	2,500
10	MeOH	TFA	60	2	AWP C8	6	6	6	3,104	15	9	15,992

Exp No	MP	ION	TEMP °C	VOL µl	COL	RAMP A min.	RAMP B min.	RAMP C min.	R_1	N	nB	K'
11	ACN	AF	50	10	BEH C4	2	10	6	0,162	7	3	1,606
12	ACN	AF	50	2	BEH C4	10	10	6	0,648	7	2	2,568
13	MeOH	TFA	60	6	AWP C8	10	10	6	3,370	13	8	23,554
14	MeOH	AF	70	2	AWP C8	2	2	10	0,632	9	2	6,036
15	MeOH	TFA	50	6	BEH C4	10	2	10	1,026	12	5	8,483
16	ACN	AF	60	10	BEH C4	6	6	10	0,601	7	2	1,277
17	MeOH	AF	50	6	BEH C4	10	6	10	0,001	9	1	8,747
18	MeOH	TFA	60	2	BEH C4	2	10	10	3,453	16	11	5,592
19	ACN	TFA	50	6	AWP C8	6	10	10	5,189	15	10	6,171
20	ACN	TFA	70	10	AWP C8	10	10	10	7,083	15	11	6,559
21	MeOH	TFA	70	10	AWP C8	10	10	10	4,459	15	9	20,251
22	ACN	TFA	60	6	BEH C4	2	2	2	2,360	11	4	2,613
23	ACN	AF	50	2	AWP C8	6	2	2	2,013	8	3	3,293
24	MeOH	TFA	50	10	AWP C8	2	6	2	2,211	15	7	10,886
25	ACN	TFA	70	2	BEH C4	10	6	2	7,696	13	9	3,499
26	MeOH	AF	70	6	BEH C4	6	10	2	0,001	8	1	5,585
27	MeOH	AF	60	10	AWP C8	10	10	2	0,985	10	2	17,141
28	MeOH	TFA	70	10	BEH C4	6	2	6	1,799	14	8	5,887
29	ACN	AF	60	10	AWP C8	10	2	6	0,613	7	2	3,523
30	ACN	AF	70	6	AWP C8	2	6	6	0,001	5	1	2,502
31	MeOH	TFA	60	2	AWP C8	6	6	6	3,129	15	9	15,835
32	ACN	AF	50	10	BEH C4	2	10	6	0,164	7	3	1,605
33	ACN	AF	50	2	BEH C4	10	10	6	0,755	7	2	2,398
34	MeOH	TFA	60	6	AWP C8	10	10	6	3,340	13	8	23,550
35	MeOH	AF	70	2	AWP C8	2	2	10	0,316	11	4	6,054
36	MeOH	TFA	50	6	BEH C4	10	2	10	1,005	12	5	8,805

Exp No	MP	ION	TEMP °C	VOL µl	COL	RAMP A min.	RAMP B min.	RAMP C min.	R_1	N	nB	K'
37	ACN	AF	60	10	BEH C4	6	6	10	0,776	7	2	1,685
38	MeOH	AF	50	6	BEH C4	10	6	10	0,001	9	1	8,612
39	MeOH	TFA	60	2	BEH C4	2	10	10	3,542	16	11	5,564
40	ACN	TFA	50	6	AWP C8	6	10	10	5,176	15	10	5,999
41	ACN	TFA	70	10	AWP C8	10	10	10	7,088	15	11	6,527
42	MeOH	TFA	70	10	AWP C8	10	10	10	4,422	15	9	20,316

Table S 2 fractional factorial experimental design (DOE_2)

Exp No	RAMP B min.	%A %	%B %	Flow ml/min	ION	N	nB	K'	R_1
1	10	20	40	0.3	PFPA	11	5	25,000	1,576
2	15	20	40	0.3	TFA	20	13	22,012	2,698
3	10	30	40	0.3	TFA	20	13	10,681	3,125
4	15	30	40	0.3	PFPA	11	5	32,206	1,66
5	10	20	50	0.3	TFA	17	10	14,829	2,08
6	15	20	50	0.3	PFPA	16	10	29,010	4,612
7	10	30	50	0.3	PFPA	14	8	13,281	2,357
8	15	30	50	0.3	TFA	19	12	10,534	2,761
9	10	20	40	0.5	TFA	19	12	28,021	3,194
10	15	20	40	0.5	PFPA	11	5	52,354	2,364
11	10	30	40	0.5	PFPA	11	5	40,449	2,323
12	15	30	40	0.5	TFA	22	15	17,615	6,714
13	10	20	50	0.5	PFPA	16	10	35,528	5,458
14	15	20	50	0.5	TFA	20	13	28,279	3,186
15	10	30	50	0.5	TFA	20	13	15,690	2,996
16	15	30	50	0.5	PFPA	16	10	37,907	1,22

Exp No	RAMP B	%A	%B	Flow	ION	N	nB	K'	R_1
	min.	%	%	ml/min					
17	10	20	40	0.3	PFPA	11	5	24,620	1,569
18	15	20	40	0.3	TFA	21	14	17,615	3,338
19	10	30	40	0.3	TFA	20	13	10,735	3,337
20	15	30	40	0.3	PFPA	11	5	32,112	1,686
21	10	20	50	0.3	TFA	16	10	15,005	1,868
22	15	20	50	0.3	PFPA	16	10	29,525	6,232
23	10	30	50	0.3	PFPA	14	8	13,207	2,252
24	15	30	50	0.3	TFA	20	13	10,507	2,741
25	10	20	40	0.5	TFA	20	13	28,015	3,138
26	15	20	40	0.5	PFPA	11	5	53,285	2,294
27	10	30	40	0.5	PFPA	11	5	39,907	2,409
28	15	30	40	0.5	TFA	21	14	17,293	7,067
29	10	20	50	0.5	PFPA	16	10	33,755	5,641
30	15	20	50	0.5	TFA	21	14	28,006	3,34
31	10	30	50	0.5	TFA	21	14	15,638	3,211
32	15	30	50	0.5	PFPA	16	10	37,500	1,252

Table S 3 Column reproducibility data of three different BEH C8 resin batches

Condition-replicate	*Area Sum µV*	*Avg Area Sum*	*STDev.*	*CV%*	*Recovery %*
W/O Column – 1	*23970801*	*24101199*	*129267*	*0.5%*	*N/A*
W/O Column – 2	*24229304*				
W/O Column – 3	*24103494*				
W/ Column – 1	*22092362*	*22482043 (93%)*	*1063253*	*4.7%*	*92%*
W/ Column – 2	*21668608*				*90%*
W/ Column – 3	*23685158*				*98%*

Details of the MS characterization techniques and corresponding results.

The identity of the main protein present in the LC chromatogram, and in the correspondent LC fractions, has been determined by two different approaches based on mass spectrometry:

Average molecular weight determination by intact mass measurement with LC-MS.

Primary sequence confirmation by peptide mapping based on trypsin digestion followed by LC-MS/MS

Intact OMV proteins were separated by RP-UHPLC chromatography and average molecular weights were determined with the in line coupled mass spectrometer. Intact mass spectra were recorded, and the mass deconvolution has been done by BioPharma Finder 2.0 software. As example the intact mass determination for PorA was reported in Figure A1.

The PorA example in Figure S1 (orange peak) allowed the detection of a typical mass spectrum of an intact protein with a Gaussian distribution of charge-states. Applying deconvolution algorithm, the average molecular weight of 39,658.80 Da has been calculated, that agrees with the theoretical average MW of PorA corresponding to 39657 Da.

In Table S1 the results for all the main protein species detected in the LC chromatogram were reported, also highlighting the identity confirmation of the identified proteins after fraction collection followed by peptide mapping with trypsin digestion and LC-MS/MS analysis.

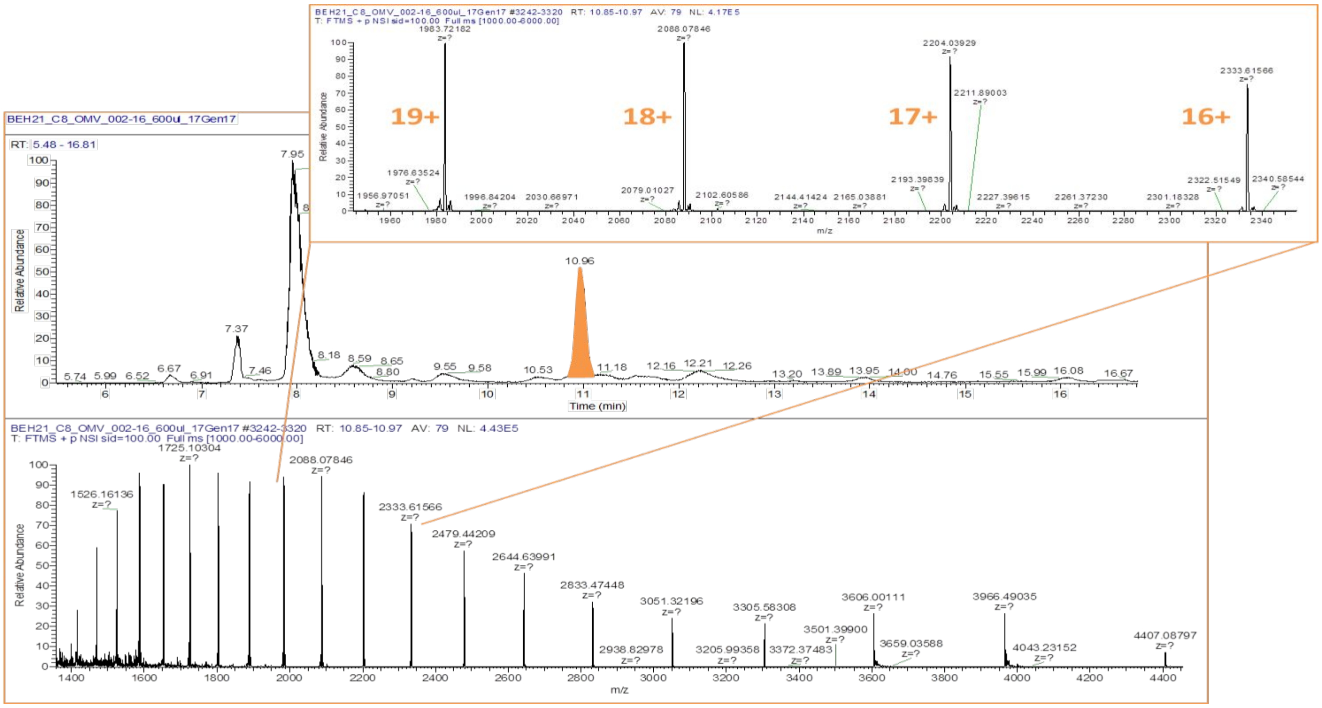

Figure S 2 Gaussian charge-states distribution of PorA protein.

Table S 4 results summary for average molecular weight determination and peptide mapping analysis for each monitored protein

**Identified protein**	**Theoretical MW (Da)**	**Detected MW (Da)**	**Identity confirmed by Peptide mapping (Y/N)**
OpcA	28007	28006.45	Yes
NspA	16521	16520.79	Yes
PorB	33721	33720.89	Yes
FetA	76729	76750.19	Yes
PorA	39657	39658.80	Yes
OmpA	23925	23921.20	Yes
FbpA	33627	33628.70	Yes
YaeT	86265	86287.42	Yes

In the following Figures (from Figure S2 to Figure S5) we report the intact mass results for the other identified proteins.

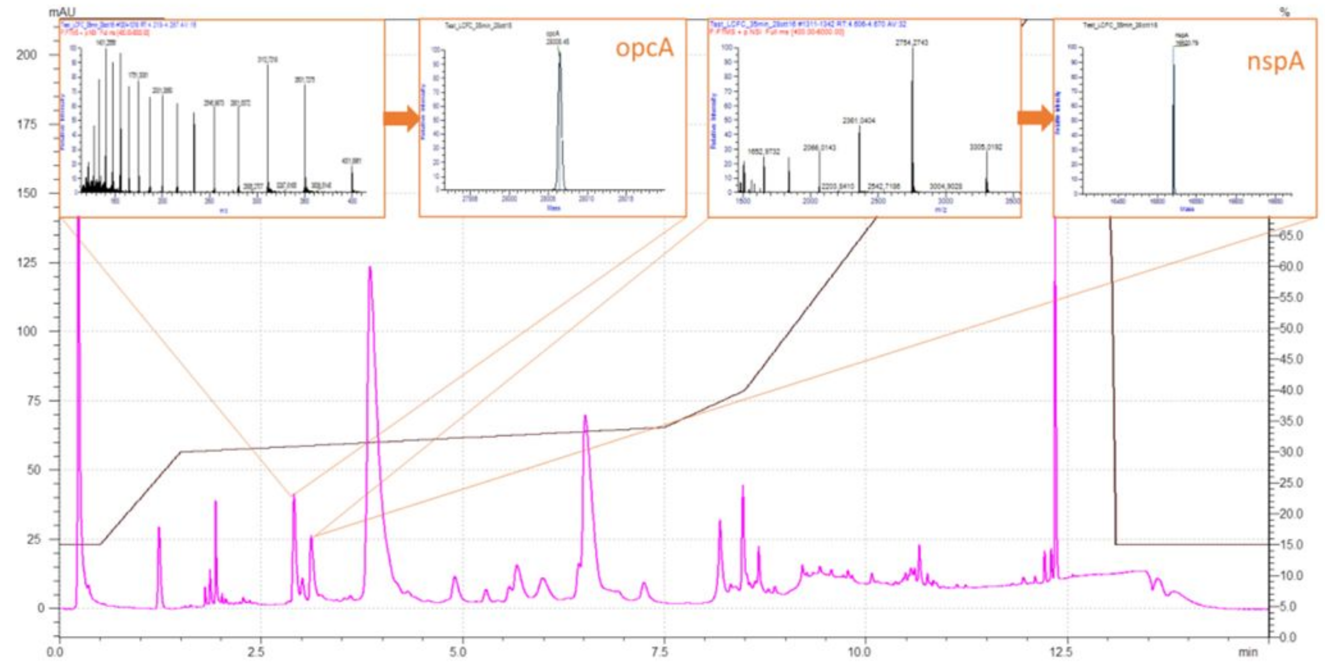

Figure S 3 Gaussian charge-states distribution of opcA and nspA proteins.

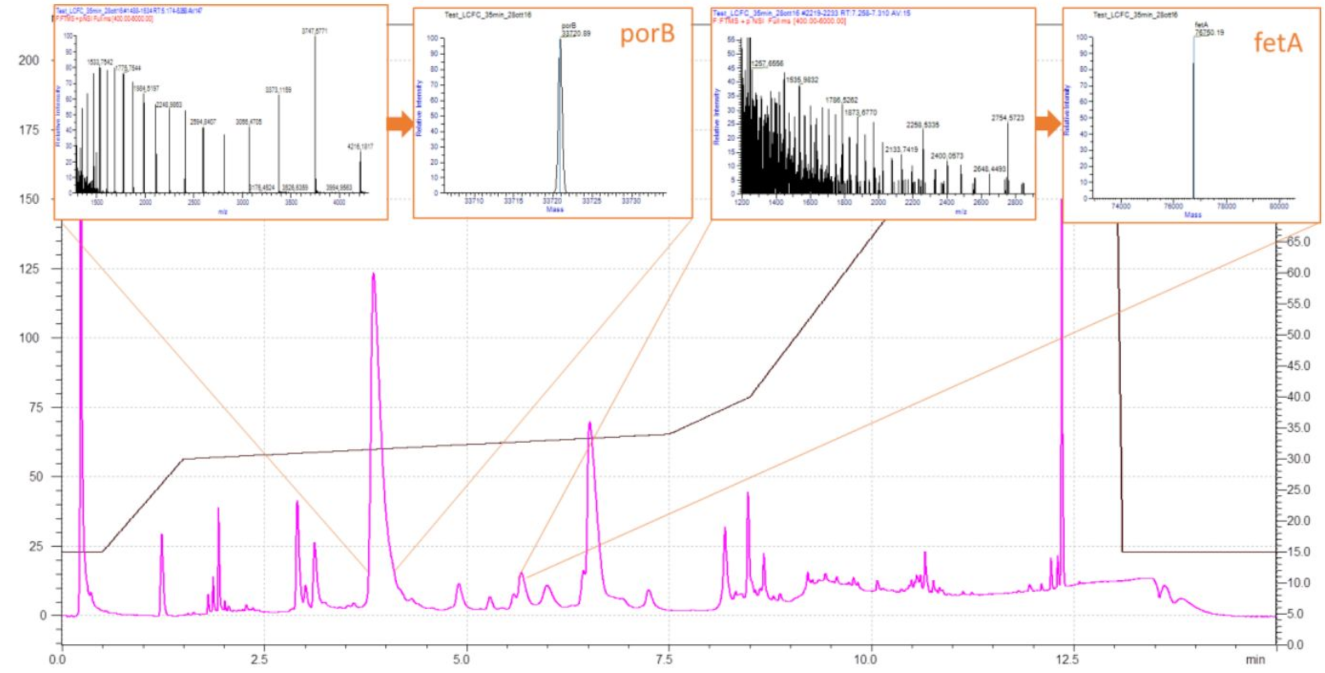

Figure S 4 Gaussian charge-states distribution of porB and fetA proteins.

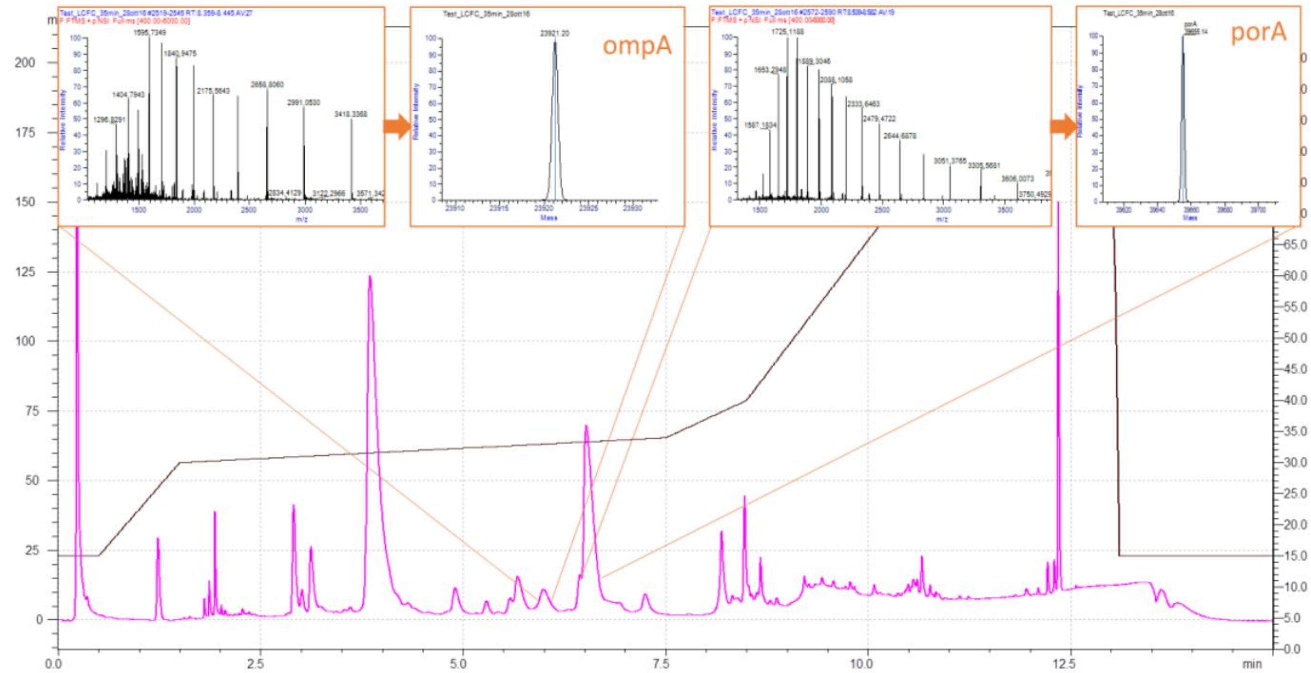

Figure S 5 Gaussian charge-states distribution of ompA and porA proteins.

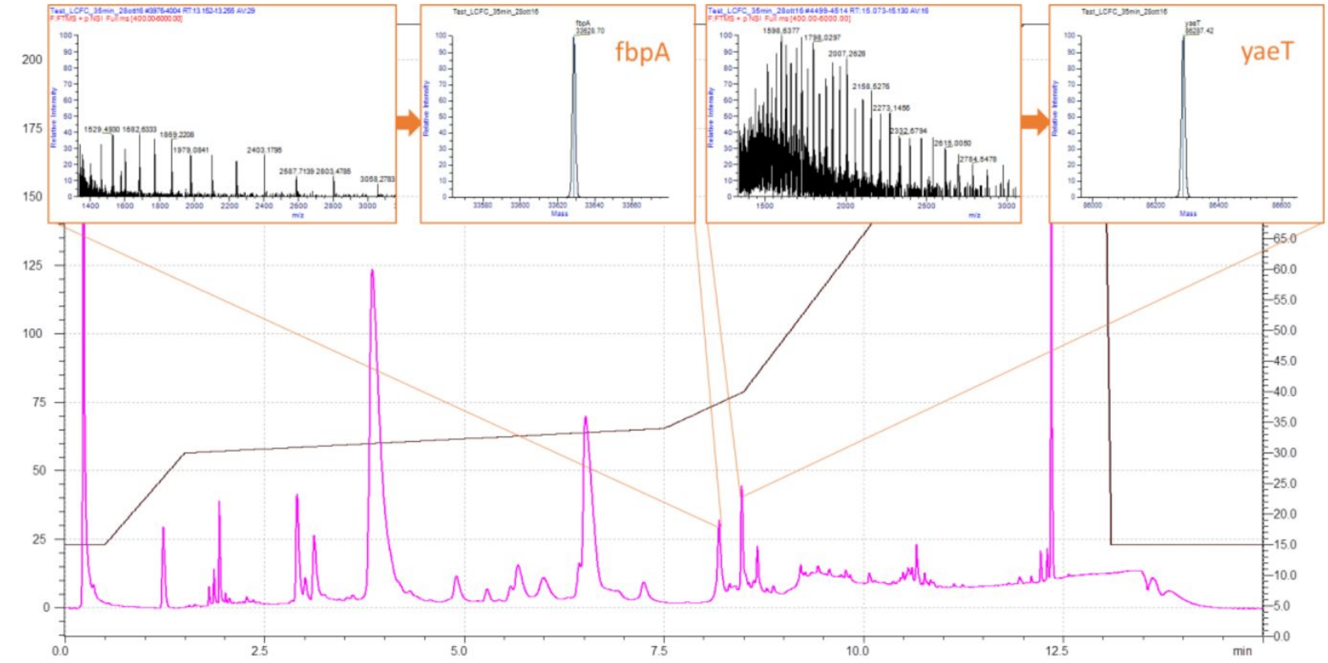

Figure S 6 Gaussian charge-states distribution of fbpA and yaeT proteins.

LC-MS analysis and peptide mapping method details

The LC-MS configurations were: Acquity H-Class Bio UHPLC system (equipped with bioQSM, bioSM-FTN and ACQ-PDA) from Waters Corp. (Milford, MA, USA) coupled with Exactive EMR system from Thermo Scientific (Waltham, MA, USA). The TriVersa NanoMate system (Advion, Inc. Ithaca, NY, USA) was adapted for LC-MS interface to reduce UHPLC flow and collect fractions.

The BioPharma Finder 2.0 software has been used for molecular weight computations and intact mass analysis of OMV /GMMA proteins.

For the MS-based peptide mapping identity confirmation, the collected fractions have undergone over N_2 evaporation process to remove the organic phase due to chromatographic elution. The dried protein fractions have been dissolved in 50 mM ammonium bicarbonate buffer, compatible with tryptic digestion, to which 0.0125 µg/µl of trypsin has been added to have a digestion of collected proteins. The samples digestion occurred overnight at 37°C. Addition of 0.1% formic acid to stop the enzymatic reaction. Samples are injected to LC-MS system to have the LC peptides separation and the fragmentation MS/MS in a Q-Orbitrap MS configuration. Over the chromatographic separation, the peptide signal is selected, filtered by the quadrupole, fragmented in collision cell, and revealed with high-resolution in Orbitrap. PEAKS Studio 8.0 software has been used for the data analysis and MS/MS spectra interpretation for the amino acidic sequences.
